# Case Report: Clinical Description of a Patient Carrying a 12.48 Mb Microdeletion Involving the 10p13–15.3 Region

**DOI:** 10.3389/fped.2021.603666

**Published:** 2021-02-25

**Authors:** Yu-qing Pan, Jian-hua Fu

**Affiliations:** Department of Pediatrics, Shengjing Hospital of China Medical University, Shenyang, China

**Keywords:** 10p deletion, copy number variation, feeding difficulty, hypocalcemia, psychomotor retardation

## Abstract

Partial deletion of 10p chromosome is a rare chromosomal aberration. Submicroscopic deletion of 10p15.3 is mainly related to cognitive deficits, speech disorders, motor delay, and hypotonia with the deleted region ranging from 0.15 to 4 Mb. The clinical phenotype is mainly determined by the *ZMYND11* and *DIP2C* genes. Here, we report a rare case of feeding difficulties, hypocalcemia, and psychomotor retardation. Our patient has a 12.48 Mb deletion in 10p15.3–10p13, which is the second case of large 10p deletion among reported cases thus far.

## Introduction

Partial deletion of chromosome 10p is a rare chromosomal aberration that is associated with different syndromes (1). It involves a known monogenic syndrome; the hypoparathyroidism, sensorineural deafness, and renal dysplasia (HDR or Barakat) syndrome (OMIM #146255); and DiGeorge syndrome 2 (DGS2), as well as other syndromes (2). HDR is a rare autosomal dominant disorder, which is characterized by varying degrees of HDR. It is caused by the dysfunction of the glutamyl-amidotransferase-subunit A (*GATA3*) gene located on 10p14 (3). *GATA3* plays an important role in the embryonic development of the parathyroid gland, inner ear, kidney, and central nervous system (4). However, haploinsufficiency of a more centromeric region on 10p13–10p14 was previously found to be related to DGS2 (velocardiofacial syndrome complex three), which also includes as a feature congenital heart defects and thymus hypoplasia/aplasia or T cell defects (5). Additional features include renal anomalies, eye anomalies, hypoparathyroidism, skeletal defects, and developmental delay (6). Importantly, hypocalcemia is one of the key characteristics present in DGS2 and HDR. Thus, this characteristic can aid in the diagnosis of the possible genetic disorders (7) (the causes of hypocalcemia are summarized in Supplementary Table 1).

In 10p13–10p15.3 microdeletions, zinc finger MYND-type containing 11 (*ZMYND11*), disco-interacting protein 2 homolog C (*DIP2C*), La ribonucleoprotein 4B (*LARP4B*), and other genes have been reported to be responsible for DGS2, HDR syndrome, or other similar phenotypes (8). The protein encoded by the *ZMYND11* gene (also called BS69 or BRAM1), a cellular nuclear protein containing PHD, Bromo, PWWP, and MYND domains, was originally identified as an adenovirus E1A-binding protein that inhibits the transactivation function of E1A (9). It is associated with autosomal dominant non-syndromic intellectual disability (or autosomal dominant mental retardation type 30, OMIM #616083) involving complex cognitive, behavioral, and developmental difficulties (10, 11). The *DIP2C* gene encodes a member of the disco-interacting protein homolog two family with two other isoforms, DIP2A and DIP2B. It is a candidate for developmental dyslexia and autism (12). The *LARP4B* gene encodes a member of an evolutionarily conserved protein family implicated in RNA metabolism and translation (13).

Recently, Kim et al. reported the so far largest deletion (16 Mb) in chromosome 10p in a Korean neonate (8). The clinical manifestations included hypoparathyroidism, hearing loss, genitourinary and cardiac anomalies, thymus hypoplasia, and neural system abnormalities and limb deformities.

In the present study, a rare case of feeding difficulties, hypocalcemia, and psychomotor retardation is reported in which our patient harbors a 12.48 Mb deletion in 10p15.3–10p13, which is the second case of large 10p deletion among reported cases so far.

## Clinical Report

**Figure 1:**
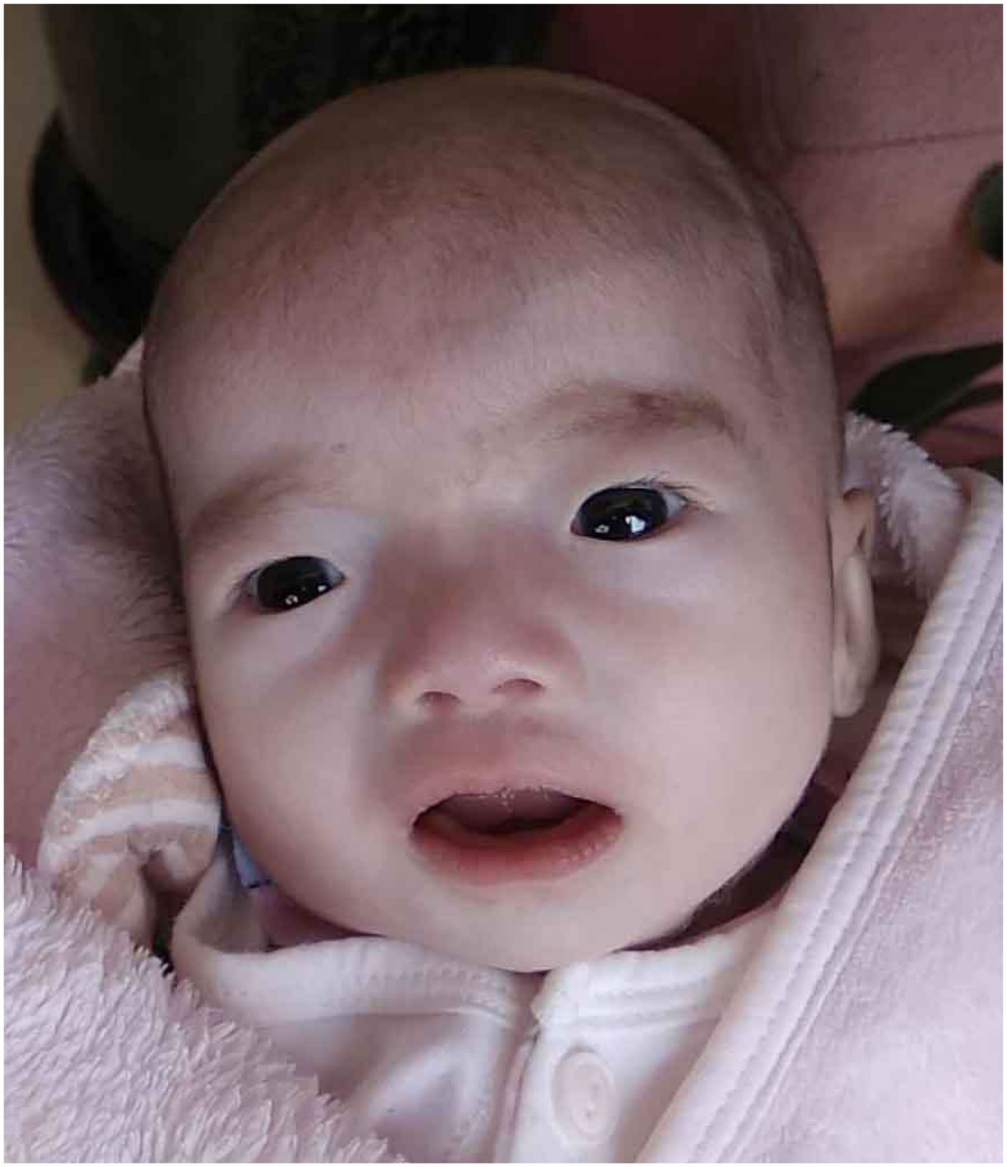
Our patient at the age of 45 days showed brachycephaly, round face, down-ward slanting palpebral fissures, hypertelorism, curled eye lashes, a broad and low nasal root, micrognathia, high arched palate, low-set ears.

Our patient was admitted when she was 6 days old to the Department of Neonatology, Shengjing Hospital of China Medical University. She was born at 39 weeks of gestation from a 34-year-old G4P2 mother without asphyxia. Her parents were non-consanguineous, and there was one healthy older female sibling in the family. The patient was spontaneously conceived, and her prenatal history was reportedly uneventful with no exposure to known teratogens. Birth weight, length, and head circumference were within normal ranges. Facial dysmorphism and feeding difficulties were noted soon after birth. Accordingly, the following features were noted: brachycephaly, round face, down-ward slanting palpebral fissures, hypertelorism, curled eye lashes, a broad and low nasal root, micrognathia, high arched palate, low-set ears, muscular hypertonia, irritability, cyanosis during feeding, weak sucking, and severe swallowing dysfunction (Figure 1). In addition, the patient had multiple daily episodes of apnea, desaturation, and cyanosis for a few seconds with spontaneous resolution.

She presented with hypoparathyroidism, and her persistent hypocalcemia was difficult to correct despite treatment with calcium gluconate solution and calcitriol. Additionally, parathyroid ultrasound did not demonstrate any anomalies, whereas a chest CT scan showed normal thymus but diffuse bilateral bronchopneumonia, and laryngotracheal CT was normal. The patient's 25-hydroxyvitamin D blood level was low (8.96 ng/ml, normal: 30–70 ng/ml), and immunological studies demonstrated normal counts and a ratio of T and B cells. Myocardial enzyme spectrum was abnormal: creatine kinase (CK) 1,335 U/L (normal: <171 U/L), CK-MB 67 U/L (normal: <24 U/L), lactate dehydrogenase (LDH) 612 U/L (normal: 80–285 U/L), and troponin I: 0.657 μg/L (normal: 0–0.04 μg/L). Myoglobin was normal. Serum creatinine and urine routine test were normal. Thus, laboratory results did not disclose renal functional abnormality. Abdominal ultrasound was planned to be performed on control examination to rule out any urogenital developmental disorder. Brain MRIs performed during hospitalization were found to be normal; however, the electroencephalogram (EEG) was severely abnormal: (1) the sleep–wake cycle was disturbed and not consistent with the corresponding gestational age; (2) EEG activity in the QS phase was abnormal with multifocal spikes and sharp waves released asynchronously; and (3) multiple θ rhythms were issued in the central midline and parietal regions. The infant's clinical manifestations and the abnormal EEG pointed toward a severe neurological dysfunction in the patient. Moreover, upper gastrointestinal radiography exhibited severe swallowing dysfunction of the epiglottis. Visual and brainstem auditory evoked potentials were observed to be normal. Fundoscopy, screening for inborn errors of metabolism, and echocardiography were found to be without a pathological sign.

## Materials and Methods

### Karyotype Analysis

G-band karyotype analysis from the patient's peripheral blood was performed (14). Karyotypes were reviewed according to the International System for Human Cytogenetics Nomenclature (ISCN 2013).

### Detection of Chromosome Copy Number Variation

Genomic DNA was extracted from the patient's peripheral blood using the DNeasy blood and tissue kit (Qiagen GmbH, Hilden, Germany). The quality and concentration of DNA were measured using the NanoDrop spectrophotometer (Thermo Fisher Scientific, Wilmington, DE, USA). Next-generation sequencing (NGS) and copy number variation (CNV) detection was performed by Berry Genomics Corporation (Beijing, China) according to previous studies (15). Briefly, 50 ng of amniocyte DNA was fragmented to an average size of 300 bp. DNA libraries were constructed by end filling, adapter ligation, and PCR amplification as previously described (16). DNA libraries were then subjected to massively parallel sequencing on the NextSeq 500 platform (Illumina, San Diego, CA, USA) in order to generate approximately five million 36-bp single-end reads, representing 0.06–0.1-fold genome coverage. All the sequences were aligned to the unmasked hg19 genome using the Burrows–Wheeler algorithm (17). Mapped reads were allocated progressively to 20 kb bin sizes from the p to q arms of the 24 chromosomes.

## Results

**Figure 2:**
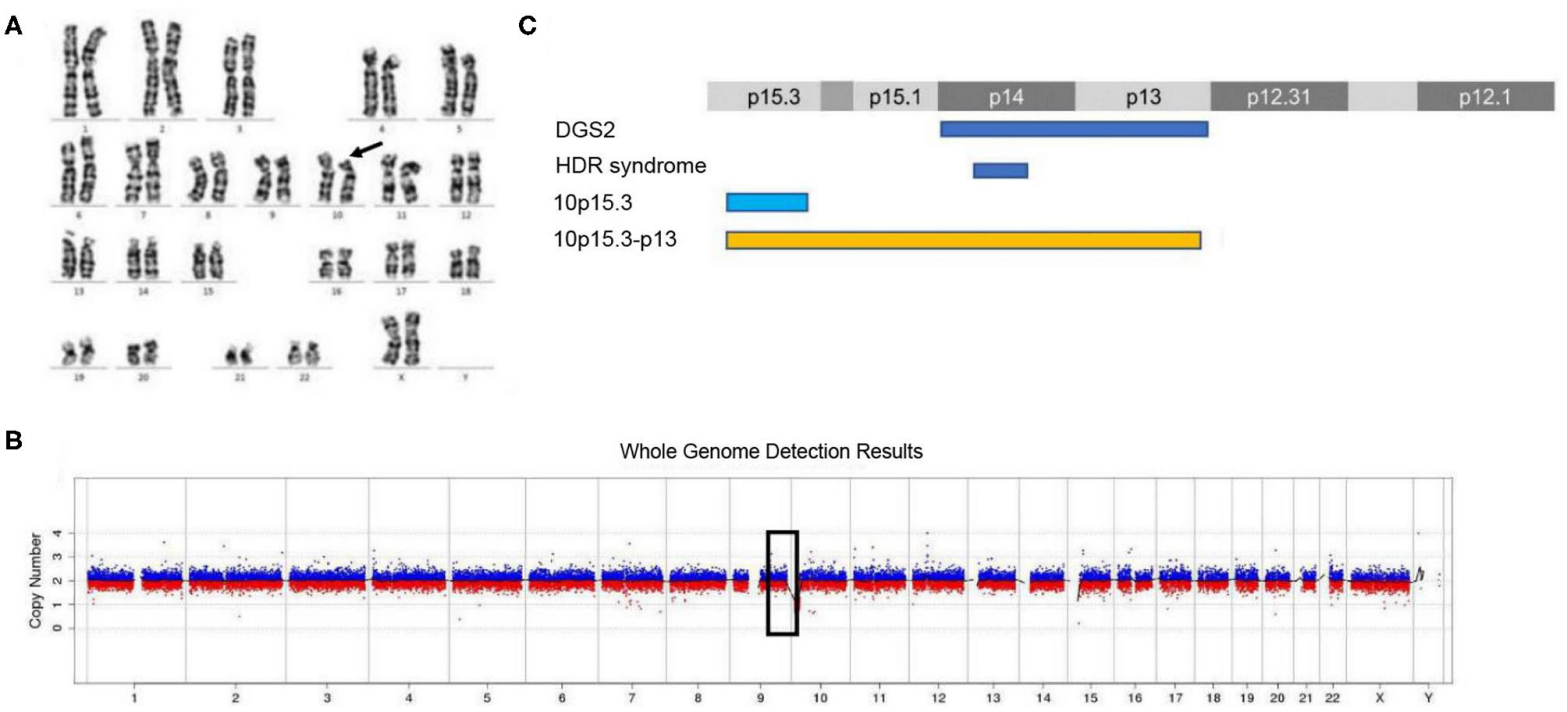
Karyotype analysis and CNV analysis showed ~12.48 Mb deletion at 10p15.3–10p13. **(A)** Karyotype analysis; **(B)** CNV sequence; **(C)** the published deletions in this region. **(C)** Showed the four deletion types in the 10p15–10p13 region. The deletion of 10p15.3 has been reported by Descipio et al. (11), Vargiami et al. (12), Eggert et al. (18), and Poluha et al. (19). The deletion of 10p15.3–p13 has been reported by Kim et al. (8) and our study.

**Figure 3:**
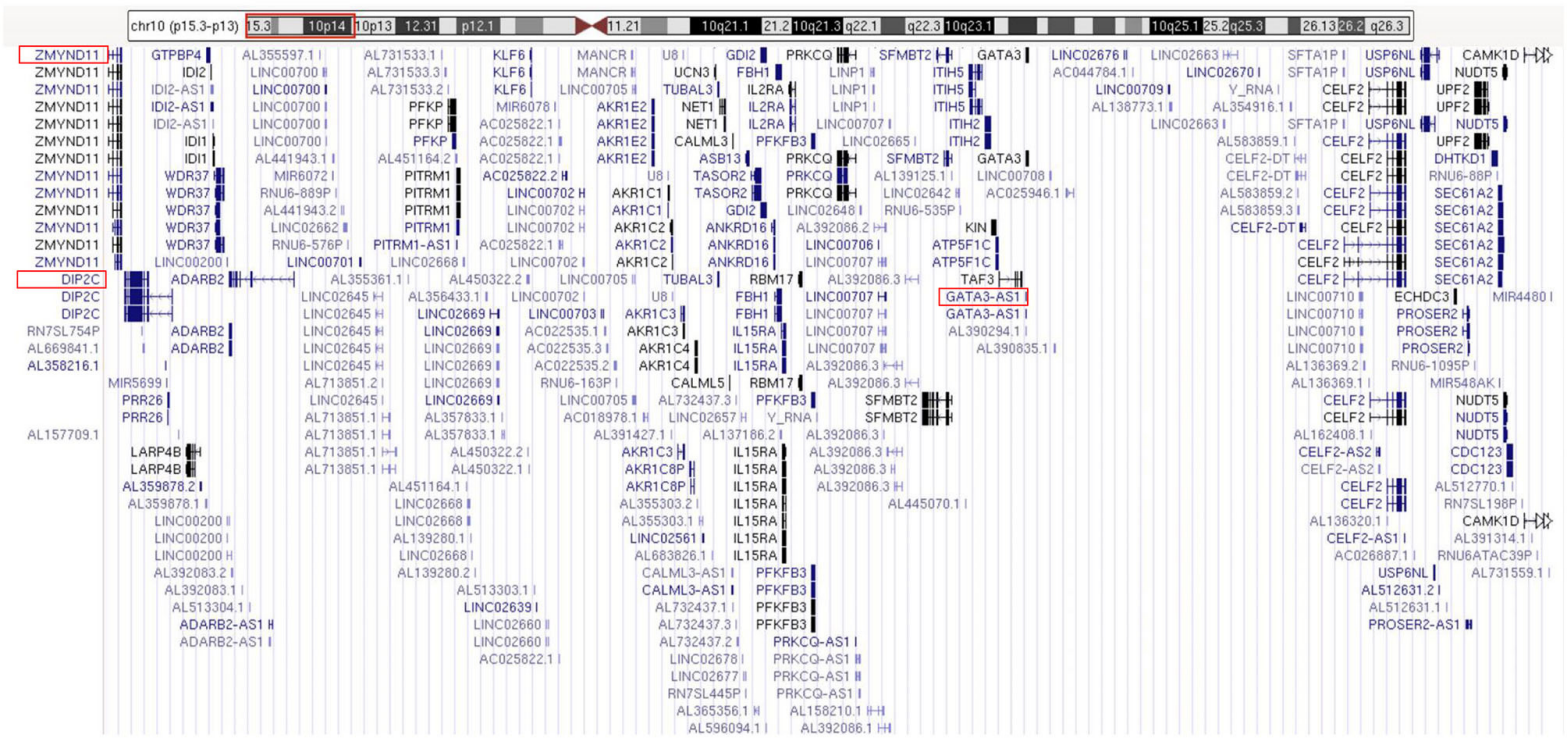
The genes included in the deleted region (chr10: 120,000–12,600,000).

The routine karyotype analysis already pointed toward a deletion in one of the short arms of chromosome 10. Subsequent NGS and CNV analyses of the data found a heterozygous 10p15.3–10p13 deletion [arr 10p15.3–p13 (120,000–12,600,000) × 1], illustrating that the patient had an ~12.48 Mb deletion in the short arm of chromosome 10 (Figure 2). The genes located in the deleted region were checked using the UCSC Genome Browser (www.genome.ucsc.edu) (Figure 3). The *ZMYND11, DIP2C, LARP4B*, and *GATA3* were included in the deleted region.

## Discussion

The clinical characteristics of reported patients with a microdeletion in the 10p13–15 region are summarized in Table 1. The present patient who has a 12.48 Mb deletion in 10p15.3–10p13 shows clinical features of facial dysmorphism, swallowing dysfunction, hypoparathyroidism, and neurological abnormalities. These phenotypes are not typical of HDR and DGS2, though the patient is only the second reported case with a large deletion.

**Table 1:** Clinical features of patients with a microdeletion/mutation involving the 10p15–10p13 region.

**Microdeletion/mutation**	**10p13–p14**		**10p15.3**				**10p15.3–p13**	
**Patient characteristics (ref.)**	**DGS2 syndrome**	**HDR syndrome**	Descipio et al. (11) (11/19 enrolled patients*)	**Vargiami et al. (12) (two patients)**	**Eggert et al. (18) (two patients)**	**Poluha et al. (19) (one patient)**	**Kim et al. (8) (one patient)**	**Our patient**
Hypoparathyroidism	n.a.	+	–	n.a.	–	n.a.	+	+
Hearing loss	n.a.	+	–	2/2	–	n.a.	+	–
Genitourinary anomalies/hypoplasia of the kidney	n.a.	+	≥1/11	–	–	n.a.	+	–
Cardiac anomalies	+	n.a.	≥2/11	–	1/2	+	+	–
Thymus hypoplasia	+	n.a.	n.a.	n.a.	n.a.	n.a.	+	–
ADHD	n.a.	n.a.	2/11	n.a.	n.a.	n.a.	n.d	n.d
Autism	n.a.	n.a.	1/11	n.a.	n.a.	n.a.	n.d	n.d
Cognitive/behavioral abnormality	n.a.	n.a.	≥10/11	2/2	2/2	+	n.d	n.d
Motor delay	n.a.	n.a.	≥10/11	2/2	2/2	+	n.d	+
Speech delay	n.a.	n.a.	≥10/11	2/2	2/2	+	n.d	n.d
Brain CT/MRI anomalies	n.a.	n.a.	≥3/11	2/2	n.a.	+	+	Normal
Facial dysmorphism	n.a.	n.a.	9/11	2/2	2/2	+	+	+
Hypertonia	n.a.	n.a.	–	+	–	+	+	+
Hypotonia	n.a.	n.a.	≥6/11	–	–	+	–	–
Seizures	n.a.	n.a.	≥3/11	–	–	+	+	+
Others	n.a.	n.a.	n.a.	n.a.	Hand/foot anomalies	n.a.	IUGR, hand/foot anomalies, hip dislocation/subluxation	n.a.

n.d meant that the symptom was not determined in the patients, n.a. meant no data available. *Descipio et al. reported 19 patients with the microdeletion, and only 11 patients have the available clinical data.

Previously, Tremblay et al. described that the deletion of chromosome 10p15.3–10p15.2 was associated with ventricular septal defects/septal aneurysms among 18 family members in three generations (20). Moreover, 19 unrelated individuals were characterized with submicroscopic deletions involving 10p15.3 (21). Their common clinical features (12 of the 19 individuals) included cognitive/behavioral/developmental difficulties, speech delay/language disorder, motor delay (10/10), craniofacial dysmorphism, hypotonia, brain anomalies, and seizures. A monozygotic female twin pair with a *de novo* 2.7 Mb deletion of 10p15.3 presented with severe developmental delay, including severe visual and sensorineural hearing impairment. Additionally, both showed generalized dystonia, microcephaly, complete absence of voluntary movements, and visual/auditory unresponsiveness (22). Submicroscopic deletion of 10p15.3 is mainly related to cognitive deficits, speech disorders, motor delay, and hypotonia with a deleted region from 0.15 to 4 Mb (21). However, Gamba et al. reported a male child with a 5.6 Mb deletion at 10p15.3–10p14 who exhibited short stature, cleft lip/palate, and feeding problems (23). In view of the aforementioned studies, the microdeletion of 10p15.3 was inferred to give rise to different features and is seemingly not related to the deletion size.

Tumiene et al. suggested that the clinical features of 10p15.3 microdeletion included neurodevelopmental disorders, characteristic dysmorphic features, and some other more frequent symptoms, and that the *ZMYND11* gene was responsible for the above phenotype (24). As a transcriptional repressor, mutations of *ZMYND11* have been associated with autosomal dominant mental retardation type 30 leading to intellectual disability, behavioral abnormalities, and seizures (25). Pathogenic single nucleotide variants (SNVs) of *ZMYND11* were also associated with Cornelia de Lange syndrome in a large study (26). In addition, the *DIP2C* gene is located on the minimal region of the overlap of the deletions (24). It is highly expressed in the brain and encompasses various neurological functions, such as “memory,” “neuropeptide signaling pathway,” and “response to amphetamine” according to a gene ontology analysis in the *DIP2C* gene knock-out mice (12). The deletion of *DIP2C* could induce the cell enlargement and growth retardation by stimulating DNA methylation (27). However, no pathogenic point mutations or gene deletions of the *DIP2C* gene have been described so far with a human phenotype. Although the *ZMYND11* and *DIP2C* genes are located on the minimal region of the overlap of the deletions (24), it seems that they serve as key genes for 10p15.3 microdeletion syndrome. However, the deletion is so large that it is impossible to correlate the entire manifestation with the function of single genes. The exact role of the *ZMYND11* and *DIP2C* genes in regard to their clinical features requires further investigation.

In summary, this study reports a patient with a 12.48 Mb deletion in 10p15.3–10p13. The phenotype was not found to be typical of HDR and DGS2, though the patient is only the second reported case having a large deletion thus far.

## Data Availability Statement

The original contributions generated for the study are included in the article/Supplementary Material, further inquiries can be directed to the corresponding author/s.

## Ethics Statement

The studies involving human participants were reviewed and approved by Ethics Committee of Shengjing hospital of China Medical University. Written informed consent to participate in this study was provided by the participants' legal guardian/next of kin. Written informed consent was obtained from the individual(s), and minor(s)' legal guardian/next of kin, for the publication of any potentially identifiable images or data included in this article.

## Author Contributions

Y-qP performed the data collection and analysis and wrote the original draft. J-hF performed the supervision and edited the writing.

## Conflict of Interest

The authors declare that the research was conducted in the absence of any commercial or financial relationships that could be construed as a potential conflict of interest.
